# Long-term exposure to lead nitrate and zinc sulfate Nile tilapia impact the *Aeromonas hydrophila* treatment

**DOI:** 10.1007/s11033-023-09033-9

**Published:** 2024-01-04

**Authors:** Ahmed H. Sherif, Lamiaa A. Okasha, Amina S Kassab, Mona E. Abass, Enas A. Kasem

**Affiliations:** 1 Fish Diseases Department, Animal Health Research Institute AHRI, Agriculture Research Center ARC, Kafrelsheikh, 12619 Egypt; 2 Bacteriology unit, Animal Health Research Institute AHRI, Agriculture Research Center ARC, Kafrelsheikh, 12619 Egypt; 3 Biochemistry unit, Animal Health Research Institute AHRI, Agriculture Research Center ARC, Kafrelsheikh, 12619 Egypt; 4 Zoology Department, Faculty of Sciences, Kafrelsheikh University, Kafrelsheikh, Egypt

**Keywords:** Lead, zinc, Liver enzymes, Cytokines, Antioxidants enzymes, Gene expression, Florfenicol

## Abstract

**Background:**

Pollution with heavy metals (HMs) is time- and concentration-dependent. Lead and zinc pollute the aquatic environment, causing severe health issues in aquatic animals.

**Materials and methods:**

Nile tilapia, the predominant cultured fish in Egypt, were experimentally exposed to 10% of LC_50_ of lead nitrate (PbNO_3_) and zinc sulfate (ZnSO_4_). Samples were collected in three different periods, 4, 6, and 8 weeks, in addition to a trial to treat the experimental fish infected with *Aeromonas hydrophila*, with an antibiotic (florfenicol).

**Results:**

Liver enzymes were linearly upsurged in a time-dependent manner in response to HMs exposure. ALT was 92.1 IU/l and AST was 82.53 IU/l after eight weeks. In the eighth week of the HMs exposure, in the hepatic tissue, the levels of glutathione peroxidase (GPx), catalase (CAT), and metallothionein (MT) were increased to 117.8 U/mg prot, 72.2 U/mg prot, and 154.5 U/mg prot, respectively. On exposure to HMs, gene expressions of some cytokines were linearly downregulated in a time-dependent manner compared to the control. After four weeks of exposure to the HMs, the oxidative burst activity (OBA) of immune cells was decreased compared to the control 9.33 and 10.3 cells, respectively. Meanwhile, the serum bactericidal activity (SBA) significantly declined to 18.5% compared to the control 32.6% after eight weeks of exposure. Clinical signs of *A. hydrophila* infection were exaggerated in polluted fish, with a mortality rate (MR) of 100%. The re-isolation rate of *A. hydrophila* was decreased in fish treated with florfenicol regardless of the pollution impacts after eight weeks of HMs exposure.

**Conclusion:**

It could be concluded that the immune suppression and oxidative stress resulting from exposure to HMs are time-dependent. Clinical signs and post-mortem lesions in polluted fish infected with *A. hydrophila* were prominent. Infected-Nile tilapia had weak responses to florfenicol treatment due to HMs exposure.

## Introduction

In recent decades, Nile tilapia (*Oreochromis niloticus)* has been the most cultured and distributed worldwide, with net production exceeding 4.5 million tons in 2020 [1]. Although Nile tilapia originated from Africa and belongs to the Cichlidae family, it is cultivated worldwide due to its high growth rate, enhanced good feed utilization, economical maintenance, and high market demand. The optimum temperature range is 25 ± 3 °C for the ideal growth performance of Nile tilapia. Also, it can tolerate a wide temperature range of 16 to 38 °C out of this range, it stops the feed intake, and mortality could be expected [1].

Environmental pollution has attracted global attention in the last decades and is becoming a severe and vital challenge for human society [1, 2]. Heavy metals released in the aquatic environment through municipal discharges wastes of agricultural industrial activity, mining, combustion of fossil fuels, and wastewater treatment plants [3], resulting in environmental pollution and raising the risk of bioaccumulation in fish tissues, threatening the life of aquatic animal, also feeding on aquatic products impacts the human health [4]. In Egypt, water polluted with heavy metals became a public health issue as domestic, agricultural, and industrial is discharged into the aquatic environment, the nature of heavy metals is toxic and could accumulate in the flesh of the aquatic animals, in turn transferred to the human body [5–9]. Heavy metals accumulate in fish organs, inducing oxidative stress [10]. The alterations occurred in antioxidant enzymes values considered oxidative stress biomarkers in fish that were exposed to environmental pollutants [11, 12]. Also, the disturbances of gene expression of metallothionein (MT) and heat shock proteins (*Hsps*) could be used as biomarkers for environmental pollutants [13, 14].

Aquatic animals that reared in the lead (Pb) polluted areas showed deleterious biochemical and physiological status [15], such as growth inhibition [16, 17], downregulating of hematological indices, initiating inflammation and apoptosis, and oxidative stress injury [18, 19]. These impacts resulted in immune depression via disturbing cytokine gene expressions [17, 18].

Zinc, a heavy metal, is widely distributed in the aquatic environment [20], it could persist for long periods in the environment and could not be biodegraded [21], causing an oxidative status and disturbing acid-base balance, damaging the gills of the aquatic animals [22]. Fish exposed to high concentrations of Zn causing structural damages adversely affect growth, development, and survival [23], accumulating in gills and leading to death by hypoxia [24, 25].

Fish exposed to sublethal concentrations of Zn showed low hatchability and survival rates, along with decreased hematological parameters and adverse alteration behaviors [26, 27]. In Egypt, Zn is commonly present in ecosystems in which Nile tilapia could be reared (fish farms) and caught (open water; Lakes, water canals, and Nile River), the concentration ranged between 0.004 and 0.46 mg/L [28], whereas Abdel-Baky et al. [29] found Zn concentration of 7.94 mg/L.

This work gives insight into treating of bacterial infection in Nile tilapia during exposure to a mixture of lead nitrate (PbNO_3_) and zinc sulfate (ZnSO_4_) pollution.

## Materials and methods

### Investigation sites

Three hundred Nile tilapia were purchased from a private freshwater fish farm at village Tolompate 7 in Kafrelsheikh governorate, Egypt. The collected fish were tranquilized in the fish farm using MS-222 (SyncaineR, Syndel, Canada) at a dose of 40 mg/L, and then the fish were transferred live to the wet laboratory of the Animal Health Research Institute at Kafrelsheikh. The acclimatization process was performed according to the recommendations [30–32]. On arrival, fish were subjected to an iodine bath (BetadineR active ingredient povidone-iodine, 5%, Nile Company for Pharmaceuticals, Egypt) at a dose of 20 ppm/L before stocking in a fiberglass tank 1.5 × 1.5 × 1 m for fourteen days, at the end of the Acclimatization period fish restored regular feeding behavioral. Two hundred and forty healthy fish were used in the experimental investigation. Water samples were collected along with the collected fish to detect the levels of Pb and Zn. At the end of the experimental period, fish were euthanized using MS-222 at a dose of 250 mg/L, and fish were kept for ten minutes after ceasing the operculum movements [33].

### Experimental trial

In experimental exposure in the lab, Two hundred and forty Nile tilapia (30 ± 5 g b.w.) were distributed into glass aquaria containing 10% LC_50_ of lead nitrate (PbNO_3_) [34] and zinc sulfate (ZnSO_4_) [35], 14.33 mg/L and 6.398 mg/L, respectively, for 4, 6, and 8 weeks.

### Bacterial infection

At the 4, 6, and 8 weeks of stocking in polluted water, Nile tilapia were experimentally infected with *Aeromonas hydrophila* 0.3 × 10^4^ CFU, which equals 10% of bacterial LD_50_. Ten Nile tilapia from each group were randomly chosen at 4, 6, and weeks of the HMs exposure and injected via intraperitoneal (IP) route with LD_50_ (2.4 × 10^5^ CFU) of an *A. hydrophila* AHRAS2 pathogenic strain (accession number MW092007) which was isolated from mass mortality of Nile tilapia reared in fish cages, bacteria were identified by Sherif and Abuleila [36]. In addition, ten fish from the control group were injected with pure saline solution (0.65%) and were considered negative controls [37]. The infected Nile tilapia were observed for two weeks for deaths. The mortality rate (MR) was estimated as follows:

MR (%) = (number of fish deaths in a specific period ∕ total fish population during that period) × 100.

Experimental Nile tilapia challenged with *A. hydrophila* and subjected to trials of re-isolation according to the following equation:

Re-isolated (%) = (number of challenged fish harbored injected microbe in a specific period ∕ total fish population during that period) × 100.

### Examination of farm and experimental fish

Following the recommendations of Amlacher [38], the experimental Nile tilapia were clinically examined for abnormal signs such as exophthalmia, dentated fines, skin hemorrhages, and detached scales, in addition to examination of the internal organs for changes in color, size, hemorrhages.

#### Heavy metals levels

The concentrations of lead (Pb) and zinc (Zn) in the experimental fish were detected in different body tissues. The collected fish were washed with distilled water, put in clean plastic bags, and stored frozen until analysis was carried out according to procedures recommended by A.O.A.C. [39]. Heavy metal analysis was carried out for water, sediment, and fish tissues according to APHA [40] using an atomic absorption spectrophotometer (Thermo electron corporations series AA Spectrometer).

#### Liver enzymes and Metallothionein

Liver enzymes, the experimental fish blood was collected from the tail vein and centrifuged to obtain sera, which were used to colorimetrically determine aspartate amino transaminase (AST) and alanine amino transaminase (ALT) using a spectrophotometer according to Reitman and Frankel [41].

Metallothionein (MT) content in hepatic tissues of the experimental Nile tilapia was spectrophotometrically measured following the method described by Derango and Page [42].

#### Cytokines gene expression using quantitative RT-PCR

The impact of water pollution on the expression of immune-related genes was assessed using RT-PCR. From the hepatic tissues, the RNA was extracted with Trizol reagent (iNtRON Biotechnology Inc., Korea), and samples were collected from three Nile tilapia at 0, 4, 6, and 8 weeks of exposure to PbNO_3_ and ZnSO_4_ and by using Nanodrop D-1000 spectrophotometer (NanoDrop Technologies Inc., USA) the obtained RNA was evaluated for its quality and quantity the kept at − 80 °C.

The complementary DNA (cDNA) was formed with SensiFAST cDNA kits (Bioline, USA) for interleukin *(IL)-1β*, tumor necrosis factor *(TNF)-β*, transforming growth factor *(TGF)-β2*, insulin-like growth factor *(IGF)1*, C-type lysozyme, and heat shock protein *(Hsp)70.* Nile tilapia-specific primers are presented in Table 1; the β-actin gene was the housekeeping gene. The data of gene expressions from RT-PCR were assessed using Eq. 2^−ΔΔCT^ [43].

**Table 1:** List of the primers set

Gene name	Acc. no. (GenBank)	Sequence	Amplicon size, bp	Annealing temperature °C
*IL-1β*	KF747686.1	5- TCTTCTACAAACGCGACACC − 3 5- TCTGGAGCTGGATGTTGAAG − 3	156	53
*TNF-β*	NM_001279533.1	5-AGGGTGATCTGCGGGAATACT-3 5-GCCCAGGTAAATGGCGTTGT-3	195	57
*TGF-β2*	NM_001311314	5-GCTCACGATCTTCCGTCTTC-3 5-CACTCCCCCTCTGTTTGTGT-3	150	57
*IGF1*	XM_003448059	5-TCTTCAAGAGTGCGATGTGC − 3 5-GGCCATAGCCTGTTGGTTTA-3	189	59
*C-type lysozyme*	LC012581.1	5-ATAAATAGCCGCTGGTGGTG-3 5-ACGACATCCGGACAAATAGG-3	207	55
*Hsp70*	FJ207463.1	5-ACAGACACCGAGAGGCTCAT-3 5-GATCTCCTCGGGGTAGAAGG-3	225	55
*β-actin*	EU887951.1	5-CCACACAGTGCCCATCTACGA-3 5-CCACGCTCTGTCAGGATCTTCA-3	111	59

**Note**: *IL-1β*: Interleukin-1 beta; *TNF-β*: tumour necrosis factor beta; *Hsp70*: heat shock protein 70

#### Antioxidants enzymes activity

Glutathione peroxidase (GPx) (EC 1.11.1.9) activity in the hepatic tissue of the experimental Nile tilapia was calculated using Mohandas et al. [44] method. The ingredients of the reaction mixture were 1.44 ml of 0.05 M PBS (pH 7.0), 0.1 ml of 1 mM EDTA, 0.1 mM sodium azide, 0.05 ml of glutathione reductase (GR; 1U/ml), 0.1 ml of 1 mM glutathione (GSH), 0.1 ml of 2 mM NADPH, 0.01 ml of 0.25 mM H2O2 and 0.1 ml of 10% PMS in a total volume of 2 ml. The disappearance of NADPH was spectrophotometrically recorded at 340 nm. Enzyme activity was calculated as nmol NADP reduced/min/mg protein using a molar extinction coefficient of 6.22 × 103/M/cm.

Catalase CAT (EC 1.11.1.6) activity in hepatic tissue of the experimental Nile tilapia was measured spectrophotometrically at 240 nm and measured as µmol H2O2 decomposed/ mg protein/min according to a method described by Lartillot et al. [45].

#### Immune assay

- **Serum bactericidal activity (SBA)**.
  The SBA of the experimental Nile tilapia was calculated following the methods developed by Kajita et al. [46]. Briefly, equal volumes of *A. hydrophila* bacterial suspension containing 1 × 10^6^ CFU/mL and experimental fish serum of about 100 µL were mixed parallel with the blank in which sterile phosphate-buffered saline (PBS) was used instead of fish serum. The ten-fold serial dilutions were made and incubated at 37 °C/24 h, and then bacterial colonies were counted on nutrient agar plates.
- **Neutrophil glass-adhesion**.
  Oxidative burst activity (OBA) of the experimental Nile tilapia was calculated following the method of Jang et al. [47], using nitro blue tetrazolium (NBT) test. In 96-well, leukocyte suspension (50 µL containing 50 cells) previously prepared from the blood of the experimental fish was loaded and incubated at 30 °C/1 h, then wells were rinsed with PBS and put at room temperature for one hour. After that, 50 µL of NBT was added to each well and fixed by methanol (30%), then left for five minutes. The wells were dried by filling with 60 µL of 2 mM potassium hydroxide and 70 µL of dimethyl sulfoxide. A plate reader performed the measures at 540 nm.

### Statistical examination of the obtained values

The pollution impacts on Nile tilapia health were statistically analyzed by the analysis of variance between different periods of exposure using one-way ANOVA and the significance of differences between means by using Duncan’s Multiple Range at a level of 0.05 [48].

### The biosafety procedures used in the experiment

All dead fish and remaining fish after the end of the experiment were burned in the fixed incinerator in the laboratory. The biosafety measures followed the pathogen regulation directorate for infectious substances (*A. hydrophila)* [49].

## Results

### The concentration of heavy metals pb and zn in the experimental fish tissues

In Table 2, Nile tilapia were subjected to Pb and Zn pollution (PbNO_3_ and ZnSO_4_ at a concentration of 14.33 mg/L and 6.398 mg/L, respectively), and Pb and Zn concentrations were measured in muscles and liver tissues at various periods. The control fish had undetectable levels of HMs, as did those exposed for four weeks. Detectable levels of Pb and Zn were detected after six and eight weeks of exposure, respectively, and the levels were associated with the time factor. Regardless of time factor, the level of Pb in liver tissues was higher compared to muscle tissues 5.33 and 6.93; 2.2 and 3.81 µg/g dry w.t., respectively. Meanwhile, Zn levels had the same trend.

**Table 2:** Level of heavy metals in experimental Nile tilapia during the experiment

Items		Pb*			Zn*	
Tissue		Muscles	Liver	Muscles		Liver
**4 weeks**	**Un-polluted**	ND	ND	ND		ND
	**Polluted**	ND	ND	ND		ND
**6 weeks**	**Un-polluted**	ND	ND	ND		ND
	**Polluted**	2.2 ± 0.2	5.33 ± 0.1	0.13 ± 0.1		4.4 ± 0.3
**8weeks**	**Un-polluted**	ND	ND	ND		ND
	**Polluted**	3.81 ± 0.3	6.93 ± 0.8	1.44 ± 0.4		5.54 ± 0.5

**Note**: *, µg/g dry body weight; W, week; ND, not detected

### Liver enzymes and antioxidants and metallothionein

**Fig. 1:**
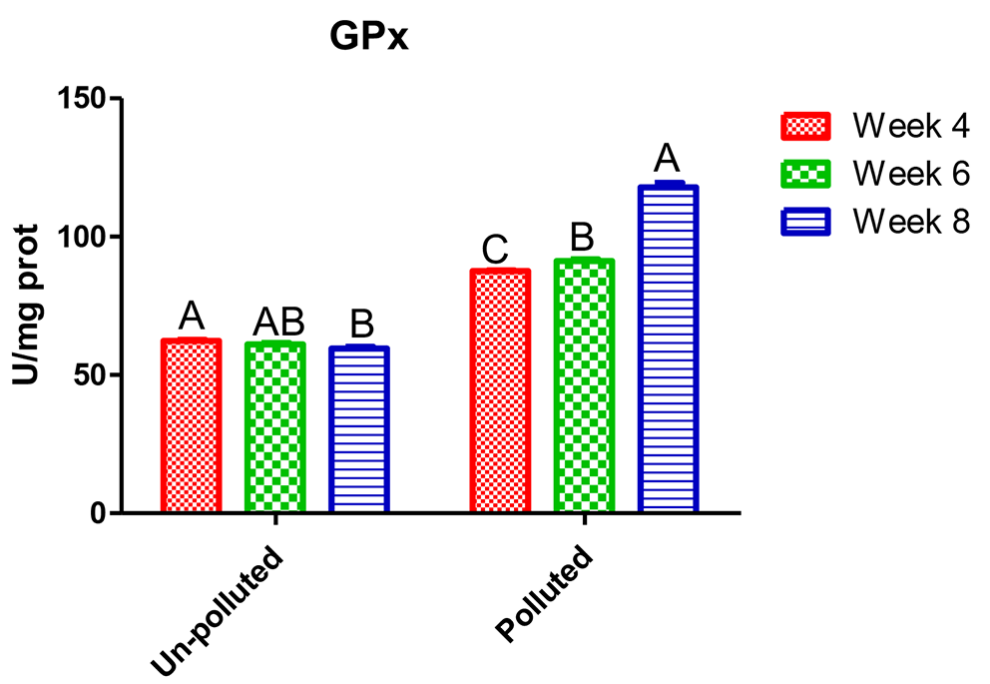
Glutathion peroxidase enzyme (GPx) level in the hepatic tissue of the experimental Nile tilapia. Different letters indicate significant difference at *P* ≤ 0.05 in time factor

**Fig. 2:**
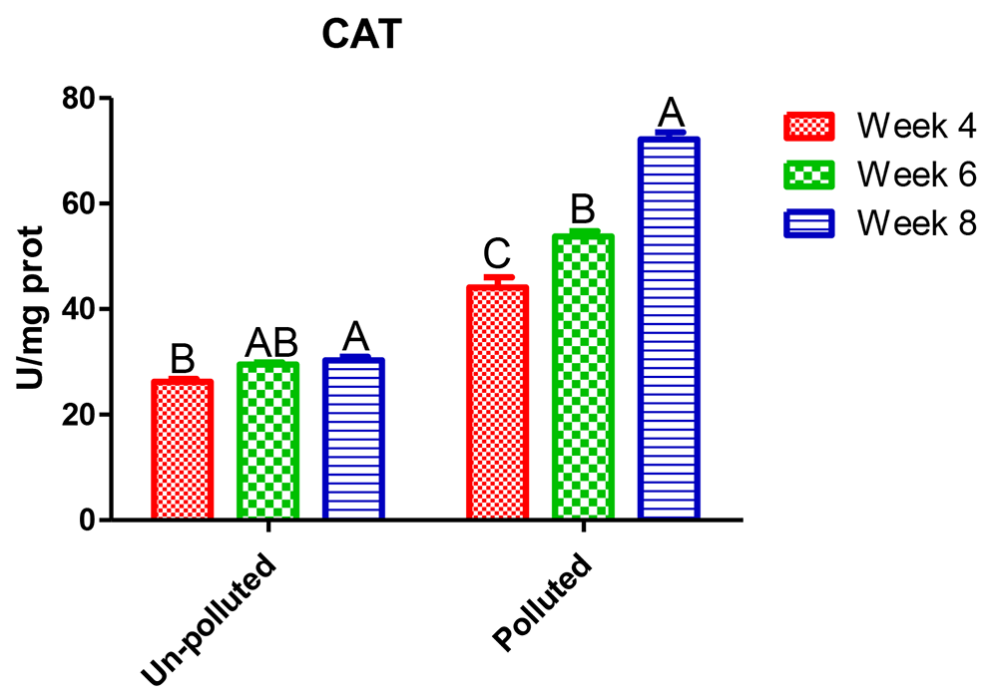
CAT level in the hepatic tissue of the experimental Nile tilapia. Different letters indicate significant difference at *P* ≤ 0.05 in time factor

**Fig. 3:**
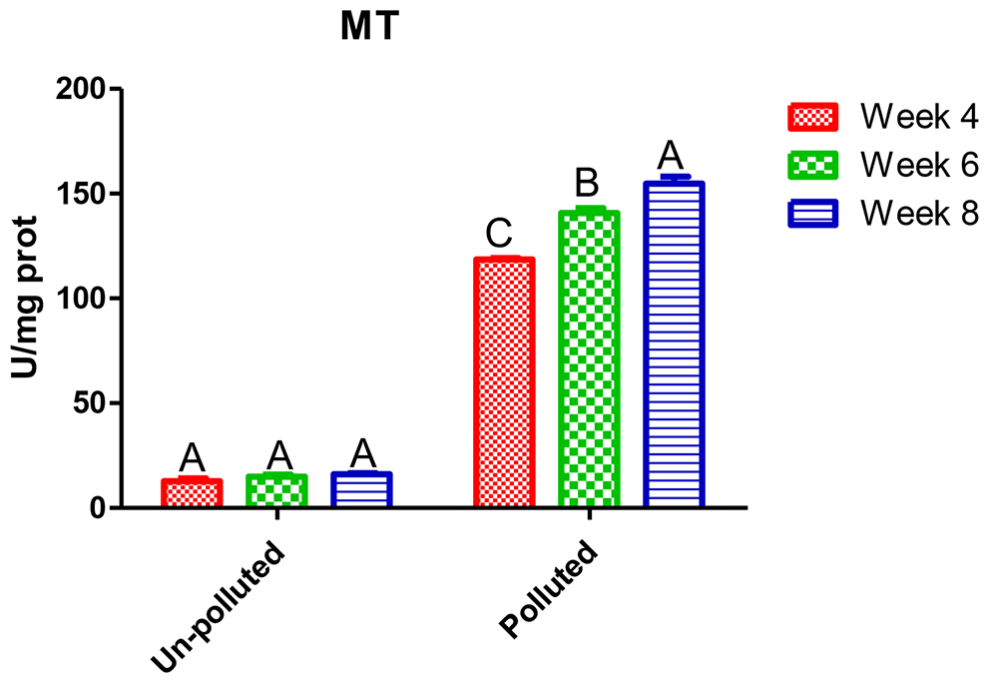
MT level in the hepatic tissue of the experimental Nile tilapia. Different letters indicate significant difference at *P* ≤ 0.05 in time factor

In Table 3, Nile tilapia exposed to a mixture of PbNO_3_ and ZnSO_4_ showed a significant deviation from normal physiological status. Liver enzymes ALT and AST showed significant and linear upsurge with time factor compared to the unpolluted group. The highest value was in the eighth week, 92.1 and 82.53 IU/l, respectively, which was about five times the control, 21.77 IU/l. On HMs exposure, antioxidants GPx (Fig. 1) and CAT (Fig. 2) had a significant rise of about 50% over the control after 4 and 6 weeks, 87.5 and 91.2; 44.1 and 53.77 U/mg prot, while their values were duplicated after eighth weeks. A ten-fold increase was observed in MT values (Fig. 3) compared to the control regardless of the time factor; the highest value was 154.5 U/mg prot after the eighth week.

**Table 3:** Liver enzymes in experimental Nile tilapia

Items	Alt IU/l		AST IU/l	
Treatment	Un-polluted	Polluted	Un-polluted	Polluted
**4 weeks**	21.83 A ± 0.93	51.4 C ± 0.71	27.8 A ± 0.61	42.87 C ± 0.77
**6 weeks**	24.5 A ± 2.5	65.47B ± 0.35	27.47 A ± 0.45	54.47B ± 0.44
**8 weeks**	21.77 A ± 1.65	92.1 A ± 1.09	30.4 A ± 0.54	81.53 A ± 0.84

**Note**: UN, undetected. Different letters indicate significant difference at *P* ≤ 0.05 in the same column

### Immunity examination

#### Gene expression of some immune related genes

**Fig. 4:**
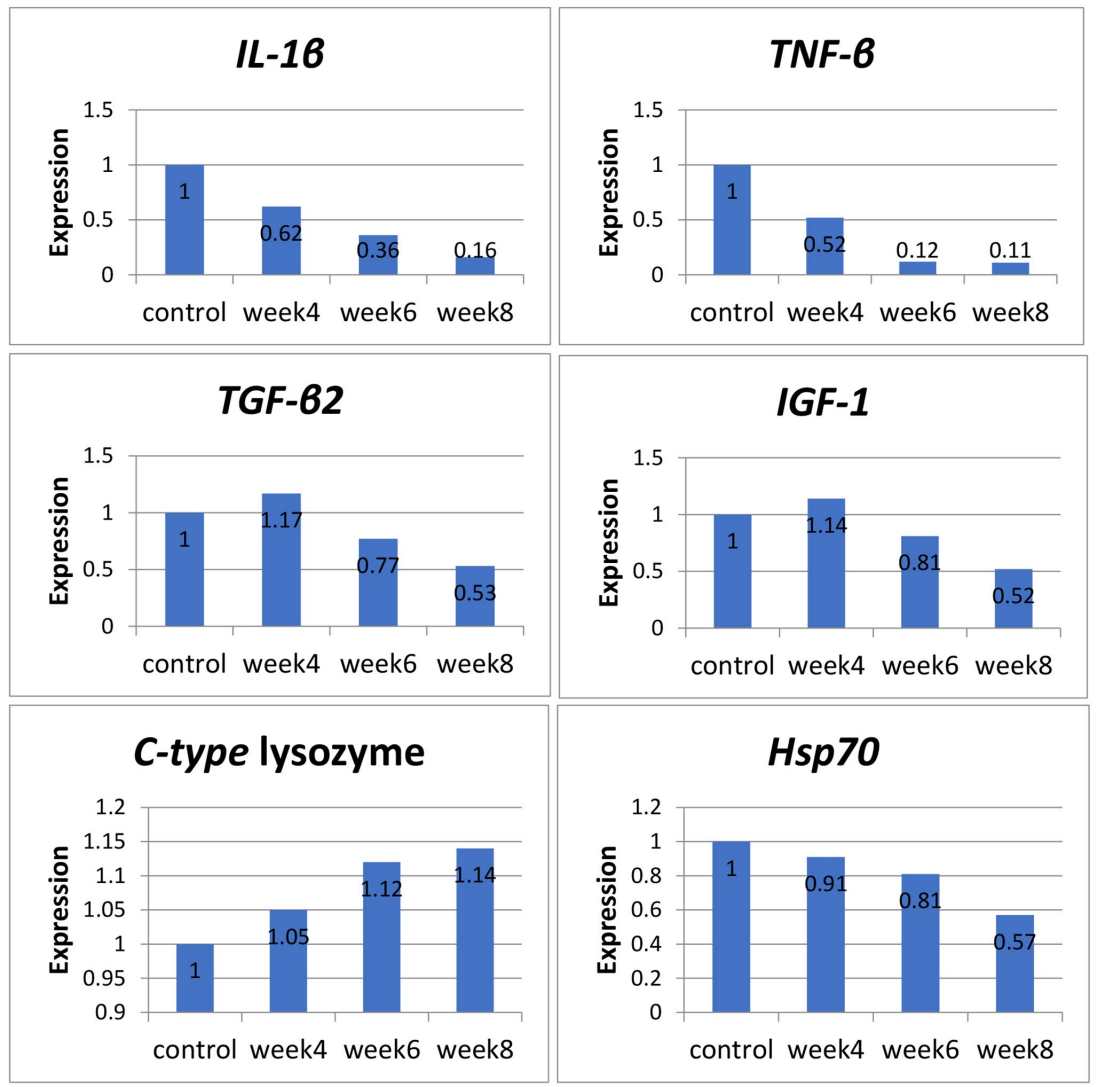
Levels of *IL-1β, TNF-β, TGF-β2, IGF1, C-type* lysozyme and *Hsp70* gene expression in relation to the expression of β-actin in liver of Nile tilapia. Different letters indicate significant difference at *P* ≤ 0.05

In Fig. 4, overall gene expression of some immune-related genes *(IL-1β, TNF-b, TGF-β2, IGF1*, C-type lysozyme, and *Hsp70)* indicated that HMs could modulate the immune status of Nile tilapia. At the same time, the expressions of *IL-1β, TNF-b*, and *Hsp70* were linearly declined with time. In contrast, C-type lysozyme increased with time and reached 1.14 fold change compared to the control 1 fold change. Whereas, *TGF-β2* and *IGF1* increased in the four week of exposure (1.17 and 1.14 fold change), then they were declined until (0.53 and 0.52 fold change) in the eighth week compared to the control (1 and 1 fold change), respectively.

#### Immune status tests

In Table 4, the activity of immune cells was examined via OBA test, Nile tilapia exposed to HMs for four weeks showed insignificant difference with the control 9.33 and 10.3 cells, whereas the activity significantly declined after 4 and 8 weeks of HMs exposure 83.3 and 5.33; 12.3 and 9.67 cells. No alterations were recorded in the SBA of Nile tilapia after 4 and 6 weeks of HMs exposure, whereas after the eighth week, a drastic decline was observed to 18.5% compared to the control 32.6%.

**Table 4:** Immunity tests in experimental Nile tilapia

Items	OBA (stained cells)		SBA (%)	
Pollution	Un-polluted	Polluted	Un-polluted	Polluted
**4 weeks**	10.3Aa ± 0.88	9.33Aa ± 0.3	28.53Ba ± 0.97	28.6Aa ± 1.13
**6 weeks**	12.3Aa ± 1.2	8.3Bb ± 0.3	29.6ABa ± 0.88	27.4Aa ± 1.3
**8 weeks**	9.67Aa ± 1.45	5.33Cb ± 0.7	32.6Aa ± 1.01	18.5Bb ± 0.8

**Note**: W. week; UN, un detected. Different letters indicate significant difference at *P* ≤ 0.05 in the same column, OBA, oxidative burst activity; SBA, serum bactericidal activity

### Mortality rate

**Fig. 5:**
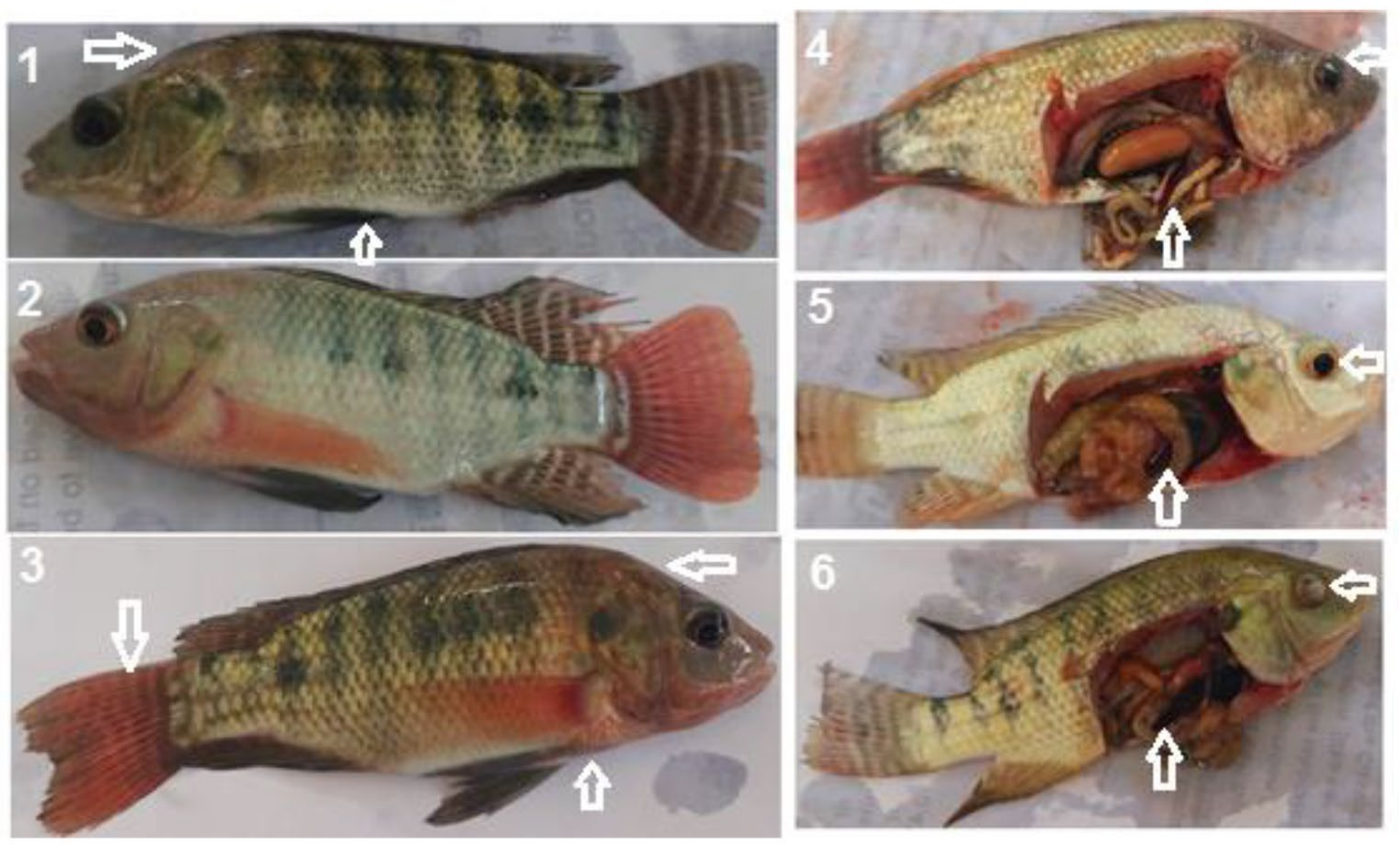
Nile tilapia challenged with LD_50_ of *A. hydrophila*. 1 external hemorrhage with sinking abdomen, 2 loss of skin pigmentation with hemorrhages on blow the mouth and fins, 3 external hemorrhages on the head, thorax and fins. 4, 5, and 6 showed splenomegaly, and eye lesions

In Fig. 5, Nile tilapia was experimentally infected with pathogenic *A. hydrophila* via intraperitoneal injection with LD_50_. The clinical signs were partially off-food, loss of body color, slight pop-eye, and hemorrhages on the skin, while post-mortem lesions were partially empty intestine and splenomegaly with different sizes. In Table 5, Nile tilapia unexposed to HMs pollution had MR ranged between 50% and 70% after experimental infection with LD_50_*A. hydrophila*, whereas for those exposed to HMs pollution, the MR was 100 after eight weeks of exposure.

**Table 5:** Mortality rate of experimental fish exposed to polluted water *A. hydrophila* infected LD_50_.

Items	Un-polluted (%)		Polluted (%)	
	Control	Infected	Control	Infected
**4 weeks**	10	50	10	60
**6 weeks**	0	60	20	60
**8 weeks**	0	70	20	100

In Table 6, after experimental infection with 10% of LD_50_ of *A. hydrophila*, MR% was higher in Nile tilapia exposed to HMs compared to the control 40% and 0 at the eighth week. Florfenicol treatment could decrease the MR to 10% in exposed Nile tilapia after six and eight weeks of HMs exposure. After exposure to HMs, the re-isolation of *A. hydrophila* was decreased in florfenicol fish compared to the untreated ones 40% and 50%; 100%, respectively.

**Fig. 6:**
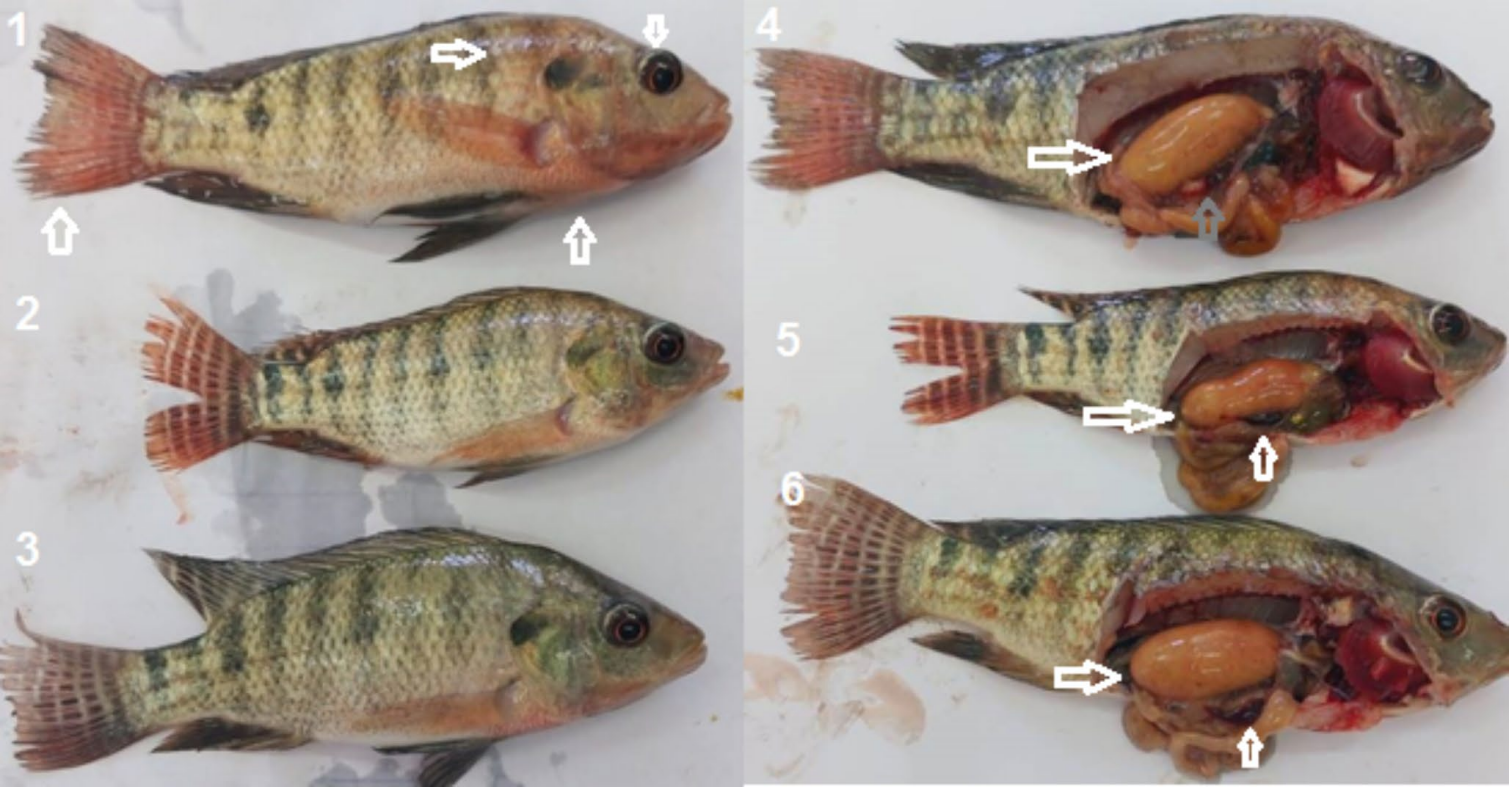
Nile tilapia infected with 10% LD_50_ of *A. hydrophila* and treated with florfenicol, in 1, 2, and 3 showed external hemorrhage, pop-eye, and dentate tail fines. 4, 5, and 6 distended intestine with food, slight splenomegaly

In Fig. 6, Nile tilapia was experimentally infected with 10% LD_50_ of *A. hydrophila.* Clinical signs were slight hemorrhages on the skin, tail dentations, and slight pop-eye. Meanwhile, post-mortem lesions were slight splenomegaly and full-intestine.

**Table 6:** Mortality rate of experimental fish exposed to polluted water 10% of *A. hydrophila* infected LD_50_.

Items	Un-polluted		Polluted	
	Control	Flor	Control	Flor
**4 weeks: MR%** **Re-isolated%**	0 20	0 20	0 50	0 40
**6 weeks: MR%** **Re-isolated%**	10 30	10 20	20 100	10 40
**8 weeks: MR%** **Re-isolated%**	0 30	10 20	40 100	10 50

**Note**: MR, mortality rate; Flor, florfenicol

## Discussion

Environmental pollution affected fish health, which could be monitored by pathological changes in the hepatic tissue [50], for example, heavy metals toxicity, which resulted in elevated liver enzymes values such as ALT and AST [51, 52]. In this investigation, long-term exposure of Nile tilapia to polluted water (PbNO_3_ and ZnSO_4_ mixture) significantly resulted in severe physiological alterations such as elevated liver enzymes ALT and AST that were about five times as the control after eight weeks. In accordance, the liver enzymes ALT and AST are very sensitive to any pathological changes in the hepatic tissue, so they could be considered the main biomarkers of body tissue damage [53–57]. Similarly, Pb toxicity could damage hepatic tissue, increasing liver enzymes in fish serum Nile tilapia [58] and common carp [59]. Accordingly, Firat and Kargin [60] found that the level of AST and ALT were increased in the serum of Zn-exposed Nile tilapia, indicating hepatic dysfunction.

Heavy metals pollution induces the production of reactive oxygen species (ROS such as superoxide, hydroxyle, and peroxides) in animal tissues, causing oxidative damage and destroying cell walls and components [61, 62], which stimulates antioxidants enzymes SOD, GPx, and CAT to counteract the propagated superoxide radicals [63]. In this work, hepatic GPx and CAT rose about double the control in Nile tilapia exposed to PbNO_3_ and ZnSO_4_ mixture. Similarly, ROS were produced in response to heavy metals bioaccumulation in fish bodies, leading to stimulate SOD, which catalytically eliminates oxidative stress [51, 64] after eight weeks. In accordance, the alterations of antioxidant enzymes are considered bioindicators for heavy metals pollution [65]; in case antioxidant enzymes cannot scavenge the propagated ROS due to Pb toxicity, cell membranes will be damaged despite upregulation of the expression of genes related to antioxidant enzymes production [66, 67].

After eight weeks of PbNO_3_ and ZnSO_4_ exposure, the hepatic MT was significantly increased compared to the control, 154.5 and 16.03 U/mg. prot, respectively. In accordance, heavy metal pollution induces the production of hepatic MT, which could be used as a biomarker, MT protects hepatic cells from being damaged by propagated ROS, decreasing oxidative stress [68]. Similarly, Zn and Cd toxicity could induce higher expression gene of MT in common carp and zebrafish (Danio rerio) [69] gudgeons (Gobio gobio) from Cd-contaminated sites in Flanders, Belgium [70]. Similarly, Darwish et al. [71] stated that MT in mammalian and non-mammalian tissues could be considered a biomarker for environmental pollution with heavy metals such as Pb and Cd.

As signaling molecules, cytokines are mediators generated by the immune cells and are necessary for the host’s defensive system [72]. In the exposed Nile tilapia, the gene expressions of *IL-1β, TNF-b*, and *Hsp70* were linearly declined in response to polluted water (PbNO_3_ and ZnSO_4_ mixture) in the hepatic tissues of Nile tilapia, indicating immunosuppression status. In accordance, alterations in the gene expressions of cytokines in the hematopoietic tissues were recorded after exposure to genotoxic compounds as HMs [73, 74]. In contrast, *Hsps* are not only induced by heat shock but also by contamination with HMs that trigger oxidative stress [70, 75]. In the experimental Nile tilapia, the expression of C-type lysozyme was linearly increased till the eighth week, whereas *TGF-β2* and *IGF1* genes were increased after four weeks of exposure and then linearly declined in the next four weeks. Accordingly, HMs pollution resulted in tissue injuries in the exposed fish [76, 77]. The decline could be due to DNA damage and immune system adaptation to long-period exposure.

In this study, there was a drastic decline in the activity of immune cells OBA and SBA of Nile tilapia reared in polluted water (PbNO_3_ and ZnSO_4_ mixture). Hence, the immune status is evaluated by assessing the serum bactericidal activity, which could disrupt bacterial cell walls, so it could be considered one of the typical methods to assess fish immunity [78]. Accordingly, ROS induces oxidative stress causing injuries and damage to the membrane of blood cells [11, 79]. Besides, fish serum possesses a bactericidal activity against Gram-positive and Gram-negative bacteria via stimulating the complement system to combat the infection [78, 80].

Our results indicated the poor health status of stressed Nile tilapia, which became vulnerable to bacterial infection [81–83]. Similarly, aquatic pollutants could impair the immune system of fish; low SBA and OBA were observed along with downregulation of *IL-1β, IL-8, TNF-α, HSP70*, and *IL-10* gene expressions [84]. In accordance, blood cells play a crucial role in fish immunity due to their phagocytic function against pathogens such as bacteria, parasites, and viruses [85], oxidative stress results in cellular damage in fish [86]. In this work, after experimental infection with 10% of LD_50_ of *A. hydrophila*, MR was higher in Nile tilapia exposed to heavy metals 40% compared to the control. Accordingly, with heavy metals exposure, Nile tilapia was susceptible to *Streptococcus agalactiae* infection, and their survivability declined [84]. Similarly, in aquatic systems, heavy metals rapidly accumulate in fish tissues, hindering their normal physiological functions and leading to high mortalities [87, 88]. In addition, Abdel-Tawwab et al. [84] found that Nile tilapia could withstand exposure to water-born Zn regimes, showing high survival rates of 96.7% and 100.0%. This result could be due to low concentration and long periods of water-born Zn exposure compared to our results.

## Conclusion

From the obtained results, it was clear that the immune-oxidative status was adversely impacted in Nile tilapia, which was exposed to lead and zinc pollution. In addition, the trial of antibiotic treatment failed to achieve its goal of eliminating the pathogenic *A. hydrophila.* In addition, the clinical signs and post-mortem lesions were amplified in polluted fish. Therefore, it is recommended that the HMs residues and immune-oxidative status should be evaluated before deciding on antibiotic treatment.

## Data Availability

Data are available on request from the corresponding author.
